# Persistence of the immune response after two doses of ChAdOx1 nCov-19 (AZD1222): 1 year of follow-up of two randomized controlled trials

**DOI:** 10.1093/cei/uxad013

**Published:** 2023-02-02

**Authors:** Merryn Voysey, Amy Flaxman, Jeremy Aboagye, Parvinder K Aley, Sandra Belij-Rammerstorfer, Sagida Bibi, Mustapha Bittaye, Federica Cappuccini, Sue Charlton, Elizabeth A Clutterbuck, Sophie Davies, Christina Dold, Nick J Edwards, Katie J Ewer, Saul N Faust, Pedro M Folegatti, Jamie Fowler, Ciaran Gilbride, Sarah C Gilbert, Leila Godfrey, Bassam Hallis, Holly E Humphries, Daniel Jenkin, Simon Kerridge, Yama F Mujadidi, Emma Plested, Maheshi N Ramasamy, Hannah Robinson, Helen Sanders, Matthew D Snape, Rinn Song, Kelly M Thomas, Marta Ulaszewska, Danielle Woods, Daniel Wright, Andrew J Pollard, Teresa Lambe

**Affiliations:** Oxford Vaccine Group, Department of Paediatrics, University of Oxford, Oxford, UK; NIHR Oxford Biomedical Research Centre, Oxford, UK; Oxford Vaccine Group, Department of Paediatrics, University of Oxford, Oxford, UK; Jenner Institute, Nuffield Department of Medicine, University of Oxford, Oxford, UK; Oxford Vaccine Group, Department of Paediatrics, University of Oxford, Oxford, UK; Oxford Vaccine Group, Department of Paediatrics, University of Oxford, Oxford, UK; Oxford Vaccine Group, Department of Paediatrics, University of Oxford, Oxford, UK; NIHR Oxford Biomedical Research Centre, Oxford, UK; Jenner Institute, Nuffield Department of Medicine, University of Oxford, Oxford, UK; Oxford Vaccine Group, Department of Paediatrics, University of Oxford, Oxford, UK; Public Health England, Porton Down, UK; Oxford Vaccine Group, Department of Paediatrics, University of Oxford, Oxford, UK; NIHR Oxford Biomedical Research Centre, Oxford, UK; Jenner Institute, Nuffield Department of Medicine, University of Oxford, Oxford, UK; Oxford Vaccine Group, Department of Paediatrics, University of Oxford, Oxford, UK; NIHR Oxford Biomedical Research Centre, Oxford, UK; Pandemic Sciences Institute, Nuffield Department of Medicine, University of Oxford, UK; Jenner Institute, Nuffield Department of Medicine, University of Oxford, Oxford, UK; NIHR Southampton Clinical Research Facility and Biomedical Research Centre, University Hospital Southampton NHS Foundation Trust and Faculty of Medicine and Institute for Life Sciences, University of Southampton, Southampton, UK; Jenner Institute, Nuffield Department of Medicine, University of Oxford, Oxford, UK; Jenner Institute, Nuffield Department of Medicine, University of Oxford, Oxford, UK; Jenner Institute, Nuffield Department of Medicine, University of Oxford, Oxford, UK; Pandemic Sciences Institute, Nuffield Department of Medicine, University of Oxford, UK; Oxford Vaccine Group, Department of Paediatrics, University of Oxford, Oxford, UK; Public Health England, Porton Down, UK; Public Health England, Porton Down, UK; Pandemic Sciences Institute, Nuffield Department of Medicine, University of Oxford, UK; Oxford Vaccine Group, Department of Paediatrics, University of Oxford, Oxford, UK; Oxford Vaccine Group, Department of Paediatrics, University of Oxford, Oxford, UK; Oxford Vaccine Group, Department of Paediatrics, University of Oxford, Oxford, UK; Oxford Vaccine Group, Department of Paediatrics, University of Oxford, Oxford, UK; Oxford Vaccine Group, Department of Paediatrics, University of Oxford, Oxford, UK; Oxford Vaccine Group, Department of Paediatrics, University of Oxford, Oxford, UK; Oxford Vaccine Group, Department of Paediatrics, University of Oxford, Oxford, UK; NIHR Oxford Biomedical Research Centre, Oxford, UK; Oxford Vaccine Group, Department of Paediatrics, University of Oxford, Oxford, UK; Public Health England, Porton Down, UK; Pandemic Sciences Institute, Nuffield Department of Medicine, University of Oxford, UK; Oxford Vaccine Group, Department of Paediatrics, University of Oxford, Oxford, UK; Jenner Institute, Nuffield Department of Medicine, University of Oxford, Oxford, UK; Oxford Vaccine Group, Department of Paediatrics, University of Oxford, Oxford, UK; NIHR Oxford Biomedical Research Centre, Oxford, UK; Oxford Vaccine Group, Department of Paediatrics, University of Oxford, Oxford, UK; Chinese Academy of Medical Science (CAMS) Oxford Institute (COI), University of Oxford, Oxford, UK

**Keywords:** vaccine, antibodies, anti-viral immunity, vaccination

## Abstract

The trajectory of immune responses following the primary dose series determines the decline in vaccine effectiveness over time. Here we report on maintenance of immune responses during the year following a two-dose schedule of ChAdOx1 nCoV-19/AZD1222, in the absence of infection, and also explore the decay of antibody after infection. Total spike-specific IgG antibody titres were lower with two low doses of ChAdOx1 nCoV-19 vaccines (two low doses) (*P* = 0.0006) than with 2 standard doses (the approved dose) or low dose followed by standard dose vaccines regimens. Longer intervals between first and second doses resulted in higher antibody titres (*P* < 0.0001); however, there was no evidence that the trajectory of antibody decay differed by interval or by vaccine dose, and the decay of IgG antibody titres followed a similar trajectory after a third dose of ChAdOx1 nCoV-19. Trends in post-infection samples were similar with an initial rapid decay in responses but good persistence of measurable responses thereafter. Extrapolation of antibody data, following two doses of ChAdOx1 nCov-19, demonstrates a slow rate of antibody decay with modelling, suggesting that antibody titres are well maintained for at least 2 years. These data suggest a persistent immune response after two doses of ChAdOx1 nCov-19 which will likely have a positive impact against serious disease and hospitalization.

## Teaser

Total spike-specific IgG antibody responses after two doses of ChAdOx1 nCov-19 (AZD1222) persist well for 1 year. Modelling suggesting titres are well maintained for at least 2 years. Therefore, two doses of ChAdOx1 nCov-19 will likely have a positive impact against serious disease and hospitalization.

## Introduction

The SARS CoV-2 pandemic continues to place a significant burden on healthcare systems and economies worldwide.

The ChAdOx1 nCoV-19 (AZD1222) vaccine is a replication deficient adenoviral vectored vaccine which encodes the SARS CoV-2 spike protein from the circulating strain first identified in late 2019. ChAdOx1 nCoV-19 has been distributed for vaccination in more than 180 countries across six continents and over 3 billion doses have been released for supply to vaccination programmes worldwide.

Vaccine efficacy (VE) against symptomatic infection in a pre-specified pooled analysis of trials conducted in the UK, South Africa, and Brazil, was 66.7% (95% CI 57·4–74·0) and in the US Phase 3 study VE was 74% (95% CI 65.3–80.5%) [1, 2] Data continue to accrue demonstrating that vaccination against COVID-19 prevents hospitalization and death with positive outcomes demonstrated after vaccination with a single dose of ChAdOx1 nCoV-19 [2–4]. Real-world data demonstrate that vaccination protects against serious disease and hospitalization following infection with variants of concern (VoC) [5, 6], and it has been estimated that the ChAdOx1 nCoV-19 vaccine has saved 6.3 million lives in the first year of the global vaccine rollout.

After peaking 4–6 weeks post-vaccination, vaccine-induced immunity, and VE against symptomatic infection begin to wane [7, 8]. The trajectory of antibody decay after a two-dose schedule has been published for some COVID-19 vaccines with 6 months of follow-up [9–12]. Antibody decay is often modelled using an exponential model with the assumption that antibody waning assumes a linear trajectory. This assumption may not hold when modelling the trajectory of antibody decay over an entire year and longer.

Currently, there are few data available on the persistence of antibody titres for one year after vaccination due to ongoing booster vaccination and virus transmission. Here we present persistence data for both antibody and T-cell responses during 1 year of follow-up after a two-dose schedule of ChAdOx1 nCoV-19, excluding those participants with a known or suspected case of COVID-19. We model the decay process to determine whether antibody continues to decay at a similar rate in the second half of the year as is observed in the first 6 months. We also investigate if decay rates vary by the dose of ChAdOx1 nCoV-19/AZD1222 vaccine received and the interval between first and second doses, and after infection.

## Materials and methods

### Participants and study procedures

Participants are from two study sites (Oxford and Southampton) from the multi-centre single-blind randomized controlled trials of ChAdOx1 nCoV-19 in the UK (COV001, NCT04324606 and COV002, NCT04400838). Participants were recruited to either safety and immunogenicity groups or efficacy cohorts and randomized to receive either ChAdOx1 nCoV-19 vaccine or a control product (MenACWY). A full description of the safety, immunogenicity (up to 28 days after second vaccination), and interim efficacy of these and related studies has been previously published, including full study protocols [2, 13, 14].Written informed consent was received from all participants.

Participants were enrolled between April and August, 2020. Participants included in this analysis received either two low-dose vaccines (LDLD) or two standard doses of vaccine (SDSD), or a low dose followed by a standard dose (LDSD) of vaccine, or three standard doses. Blood was sampled at day 90, 182, or 364 second dose (±30 days), or 28 days earlier (days 154 and 336) in those who were received two doses 28 days apart. Control group participants were not included in the analysis which is focussed on the persistence of immune responses to ChAdOx1 nCoV-19 vaccine only.

### Immunogenicity assessments

Total anti-spike IgG was measured using an in-house standardized total IgG ELISA against trimeric SARS CoV-2 spike protein. Live virus microneutralization titres were measured by Health Security Agency (Public Health England) using the Victoria strain of SARS-CoV-2 [15]. Cellular responses were assessed using an *ex vivo* interferon-γ enzyme-linked immunospot (ELISpot) assay to enumerate antigen-specific T cells [15].

Anti-ChAdOx1 vector neutralization titres were measured using a secreted embryonic alkaline phosphatase (SEAP)-reporter assay, which measures the reciprocal of the serum dilution required to reduce in-vitro expression of vector-expressed SEAP by 50%, 24 h after transduction [16].

### Statistical methods

Log-transformed anti-spike IgG data were analyzed in a mixed effects log–log regression model regressing log-transformed IgG values on log-transformed days from vaccination. Participant-level random intercepts were included along with fixed effect terms for participant age (years, centred on age 40 years), the interval between first and second doses (< 8 weeks, 9–12 weeks, >12 weeks), and the vaccine doses received (LDLD, LDSD, SDSD). Log–log models were compared with log–linear models using Akaike’s Information Criteria (AIC).

A similar approach was applied to the analysis of ELISpot and neutralization data. However, terms for interval between first and second doses, and vaccine received were not included as these were not applicable to the data, which was available on a subset of SDSD participants only and without peak immunogenicity timepoint of day 14 post primary vaccination.

Participants who had evidence of SARS-CoV-2 infections during the follow-up period were excluded from the main models, as were those who received third doses of vaccine or additional vaccines outside of trial procedures. For each participant, an arbitrary threshold of a more than 2-fold increase in antibody titres between two study visits was considered evidence of possible infection or unreported vaccinations outside the trial and these were excluded.

A separate model was fitted to antibody values from timepoints subsequent to a positive PCR test in vaccinated participants and the decay slope modelled with a similar log–log mixed effects regression model with participant-level random effects and no additional fixed effects.

Analysis was done using R version 4.1.1. R code and output are shown in the Appendix.

Ethical review was provided by the South Central Berkshire Research Ethics Committee (20/SC/0179).

## Results

There were 600 participants included in the analysis of anti-spike IgG for whom between one and four blood samples were available from timepoints post second dose of vaccine.

Over a 1-year follow-up period log–log models fit the serology data better than log–linear (exponential decay) models due to faster decay of antibody in the first half of the year than later in the year.

**Figure 1.**
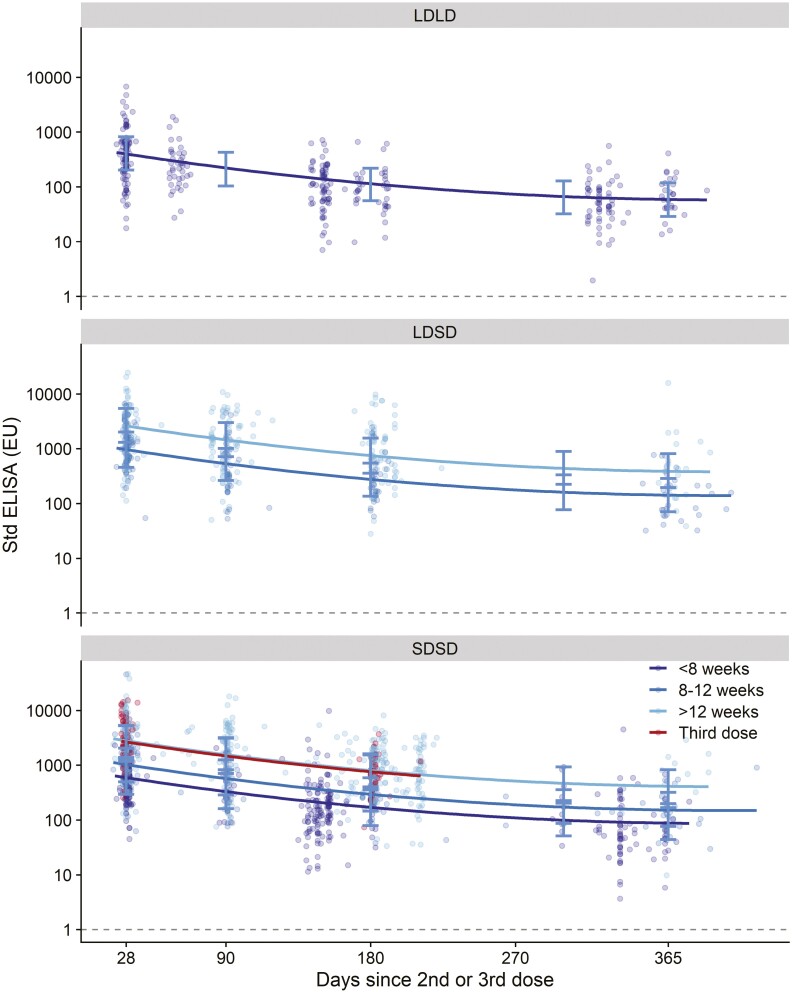
Anti-spike IgG by standardized ELISA, by vaccine and dose interval. LDLD: two low doses; LDSD: low dose followed by standard dose vaccines; SDSD: two standard dose vaccines. Each dot represents one sample. Regression lines show estimates from mixed effects regression model of log-transformed ELISA values regressed against log-transformed days since vaccination (log-log model), adjusted for age (continuous), vaccine schedule (LDLD, LDSD, SDSD) and interval (< 8 weeks; 8-12 weeks, > 12 weeks). A model testing the interaction term between days and interval was a poorer fit to the data (based on AIC) therefore the interaction was not retained and the slopes of regression lines are fixed showing parallel decay. Decay of antibody after a third dose is consistent with the pattern of decay after two doses. Dotted line shows lower limit of the assay (EU=1). Error bars show bootstrapped confidence intervals for model predicted values at day 28, 90, 180, 300 and 365 from the model output. These are also shown in Tables S1 and S2. The dataset contains 1796 samples from 704 participants

**Figure 2.**
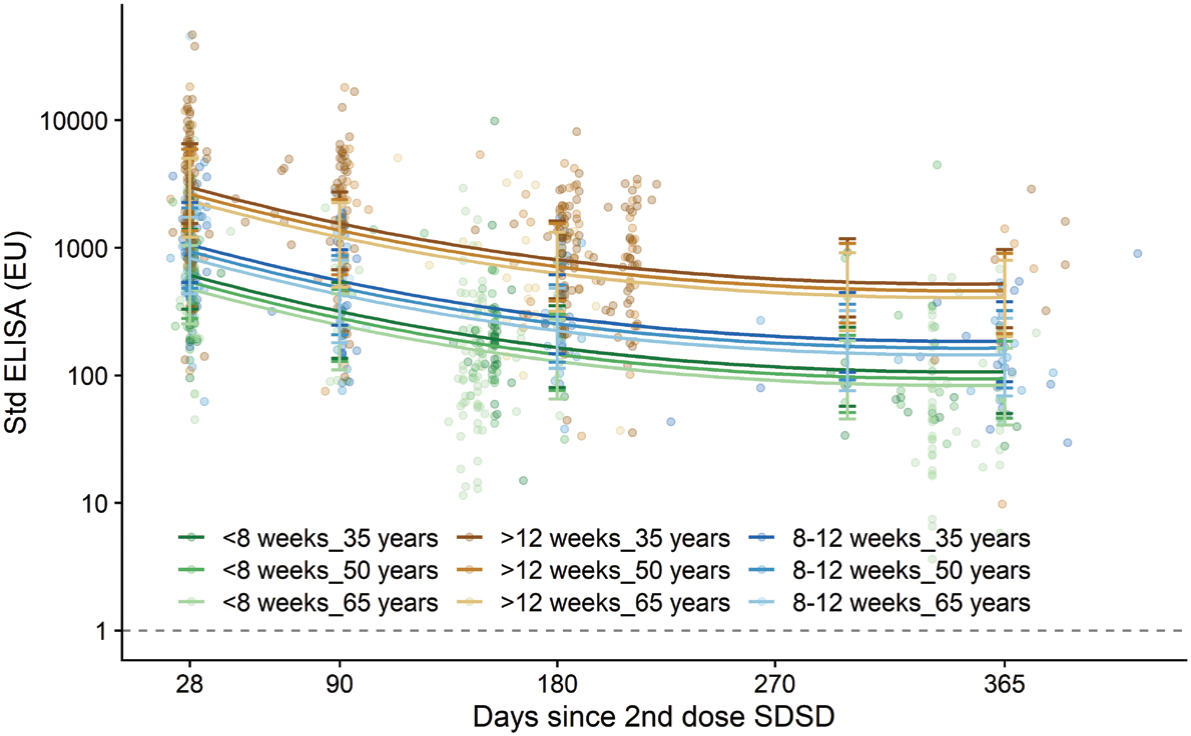
Anti-spike IgG by standardized ELISA, by age and dose interval (SDSD recipients only. SDSD: two standard dose vaccines. Each dot represents one sample. Regression lines show estimates from mixed effects model shown in figure 2 for those who received SDSD vaccines only. Age was significant in the model (P=0.0008), but the magnitude of the effect was less than the effect of the timing of the first two doses. Dotted line shows lower limit of the assay (EU=1). Error bars show bootstrapped confidence intervals for model predicted values at day 28, 90, 180, 300 and 365 from the model output. The dataset contains 1796 samples from 704 participants

Total spike-specific IgG antibody titres were lower with two low doses of ChAdOx1 nCoV-19/AZD1222 vaccines (LDLD) (*P* = 0.0006) than with SDSD or LDSD regimens (Fig. 1, Table S1). However, serological responses after SDSD and LDSD vaccines were not significantly different from each other (*P* = 0.47). Longer intervals between first and second doses resulted in higher antibody titres (*P* < 0.0001); however, there was no evidence that the decay trajectory differed by interval or by vaccine dose, and therefore the model without the interaction was retained. Older age was associated with lower titres irrespective of dose received (Fig. 2), the age by days interaction was non-significant and removed from the model (Table S1). Following a third dose of ChAdOx1 nCoV-19/AZD1222, the decay of IgG antibody titres followed a similar trajectory as that observed after two doses. No differences between males and females were observed, as previously published [17].

**Figure 3.**
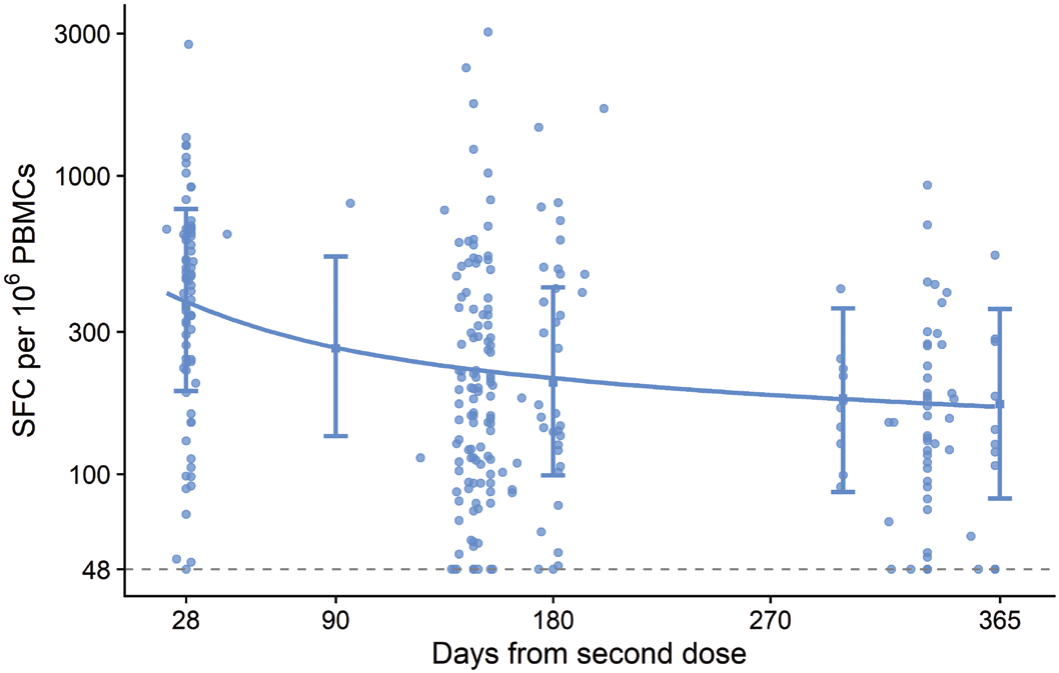
Interferon-γ ELISpot response to peptides spanning the SARS-CoV-2 spike insert in participants who received two standard doses. Each dot represents one sample. The dataset contains 292 samples from 167 participants. Dotted line shows lower limit of the assay at 48 SFC per 10^6 PBMCs. Error bars show bootstrapped 95% confidence intervals from the model output. These are also shown in Table S3

A similar modelling approach was applied to the ELISpot response (Fig. 3). Data were available from participants who received two standard doses < 8 weeks apart and there was more rapid reduction in spot forming cells (SFCs) in the first 6 months after vaccination than in subsequent time periods. Geometric mean SFCs per 10^6^ PBMCs (95% CI) were 364 (190, 773) at day 28, 203 (99, 423) at day 180, and 172 (83, 358) at day 365. (Fig. 3, Table S3). Of note, the peak in t-cell responses is generally 14 days after vaccination and so these responses represent decay from a previous higher peak at an unmeasured timepoint.

**Figure 4.**
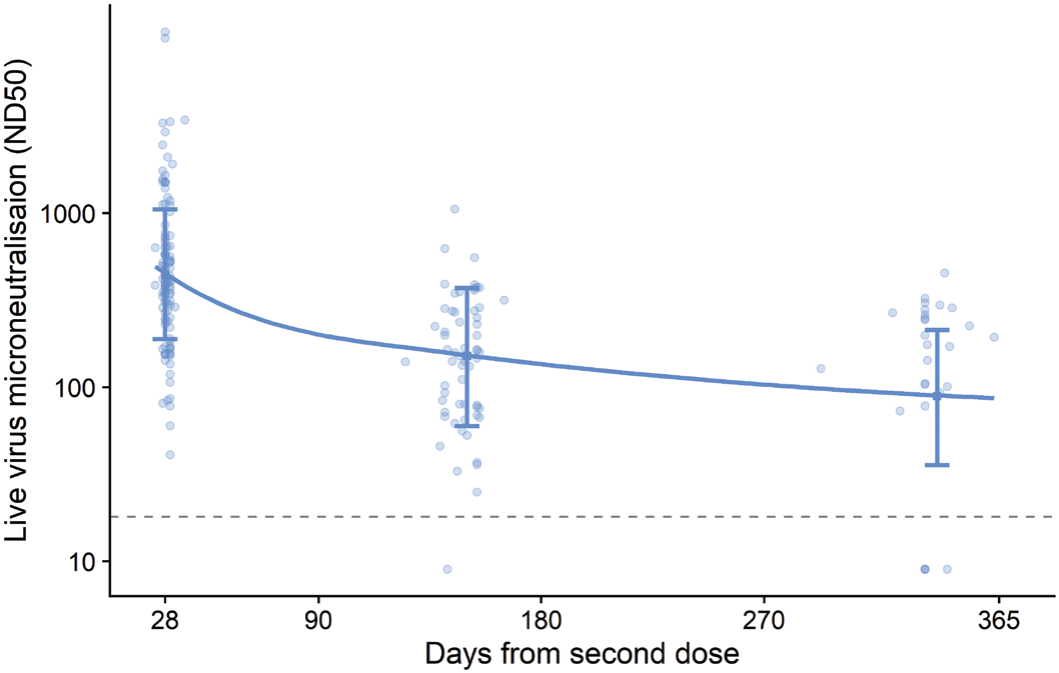
Live virus microneutralization titre (ND50) in participants who received two standard doses. ND50: Dilution to achieve 50% virus neutralization. Each dot represents one sample. Dotted line shows lower limit of the assay at a titre of 18. Error bars show bootstrapped 95% confidence intervals for model predicted values at day 28, 150, and 340 from the model output. These are also shown in Table S4. The dataset contains 198 samples from 114 participants

Limited data were available for measuring neutralization using a live microneutralization assay in participants who received SDSD regimens. Trends were similar to the analysis of both total anti-spike IgG antibody and ELISpot responses with initial decay more rapid than at later time points (Fig. 4, Table S4).

Data from assays measuring anti-ChAdOx1 vector neutralizing titre demonstrate that these titres are well maintained out to day 180 and fell from a geometric mean titre of 345 at days 28 to 203 at day 180, with substantially overlapping confidence intervals (Fig S2, Table S5).

**Figure 5.**
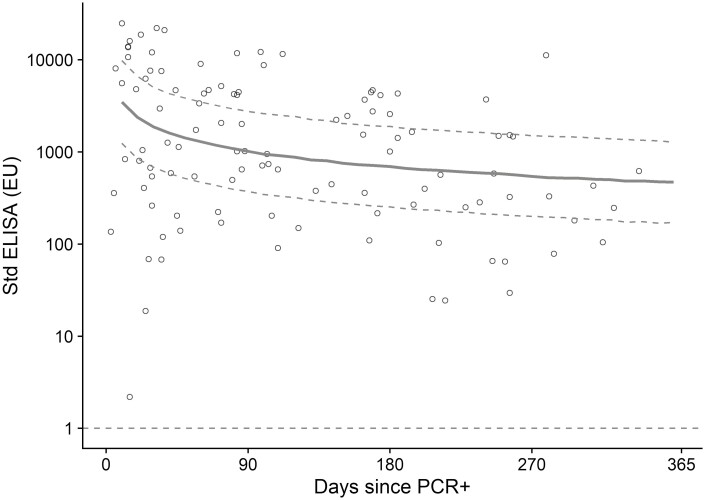
Anti-spike IgG by standardized ELISA in participants who were vaccinated and then had a positive PCR test. Data shown are ELISA values at timepoints after a positive PCR test, in participants who received two doses of vaccine prior to a positive PCR test. Each dot represents one sample. The solid line is the regression curve from a linear mixed model of log-transformed ELISA values regressed on log-transformed days since PCR test, with a participant-level random effect. Dotted curves show the bootstrapped predicted 95% confidence limits. Dotted horizontal line shows lower limit of the assay (EU=1)

Total spike-specific IgG antibody titres in vaccinated participants with positive PCR tests after vaccination showed a similar pattern of decay although fewer data points were available for robust model comparisons (Fig. 5).

## Discussion

In the absence of infection, our analysis demonstrates that waning of antibody is more rapid in the first 6 months after vaccination with ChAdOx1-nCoV-19/AZD122 than from 6 to 12 months after vaccination. Importantly, antibody waning is slower at later time periods and titres are maintained a year after vaccination with a primary series (two doses) and have a similar rate of decay in individuals who were infected after vaccination. Extrapolation of antibody levels out to 2 years post vaccination predicts levels that are well maintained. In addition, the T-cell response is well-maintained out to a year with a slow rate of decay. At a population level, the high levels of immune persistence seen will have a positive impact on serious disease and hospitalization. Not surprisingly, in those regimens that achieved higher antibody titres (two SD vaccinations in younger adults), the titres are higher at later time-points.

While mRNA-based vaccines have been shown to induce higher titre antibodies after primary series vaccination than viral vectors, there is rapid onset of antibody decay after mRNA vaccination [18]. The slow rate of immune decay after two doses of ChAdOx1 nCoV-19 is similar to that observed after one dose of Ad26 CoV-2 [18, 19]. The mechanism underpinning antibody persistence across vaccine regimens is unclear—data are accruing which suggest vaccination against COVID-19 with mixed vaccine modalities (e.g. viral vectors and RNA) results in significantly better immune persistence and a wider repertoire of antibody responses [20, 21]. A diverse and well-maintained memory response, capable of recognizing VoC, will be the preferred option to best protect individuals going forward.

UKHSA have shown rapid waning of protection from infection in the 6 months after vaccination, for all COVID-19 vaccines. Our results are consistent with some waning of efficacy in the first 6 months but between 6 and 12 months efficacy against severe disease and hospitalization may be well maintained. Antibody waning followed a similar trajectory after a primary series vaccination and after vaccination followed by infection. Sequential exposure to VoC, which will happen in periods of high virus circulation and transmission, will provide a natural boost to pre-existing immune responses against SARS CoV-2 and may augment the persistence of high titres of antibodies for sustained periods of time. Efficacy estimates can be underestimated in test-negative case control studies because it is difficult to remove all previous (and unknown) infections from the analysis, causing what is often referred to as ‘deletion of susceptibles’ bias. This is particularly problematic later in the pandemic, when an increasing proportion of unvaccinated controls is immune following prior undetected infection.

In our analysis, the interval between vaccine doses had a larger impact on the resulting antibody titres at later timepoints than the type of dose received (e.g. SD vs LD). Both real world and randomized controlled trial data from mRNA vaccine regimens also support this finding, demonstrating that antibody levels were higher after the extended dosing interval (6–14 weeks) compared with the conventional 3- to 4-week regimen [12, 22] supporting the extended vaccination interval used in the UK. Regardless, it will be important to investigate the optimum interval for booster vaccinations and while it is unclear if booster doses will be an ongoing requirement it will be important to assess in high-risk individuals, including older adults.

Third and fourth dose booster vaccination programmes have been rolled out in many countries and as such, the protection provided by two-doses of vaccines 12 months after cannot be easily determined, particularly as immunity will also be influenced by the SARS-CoV-2 variants in circulation. We have demonstrated that a third dose ChAdOx1 nCoV-19/AZD1222 can augment antibodies titres and we demonstrate here the kinetics of antibody decay following a third dose of ChAdOx1 nCoV-19 is similar to that after the primary series. Boosters may help reset the resting immune response to a higher level resulting in augmented protection against VoC and resulting in better persistence of class-switched, high affinity long-lived memory B cells.

Importantly, T-cell responses are also well maintained, and there is increasing evidence that cellular immunity and non-neutralizing antibodies are key mediators in protecting against disease caused by VoC [23, 24]. The starting point for analysis of decay of cellular responses was measured at day 28, not at the peak response time for T-cell responses of day 14. This may affect model estimates, making decay appear slower than it otherwise would if the peak response time could be observed. Real world data are demonstrating the protective impact of hybrid immunity induced post-vaccination and infection.

### Limitations

There are some limitations to these analyses. These are exploratory analyses and data were not available for the same participants for every assay as these are secondary time points. Although all participants with suspected infections or unreported vaccinations were removed from the analysis, some cases may have been missed and these participants may have antibodies that are affected by their infection.

Our data demonstrate that after a primary series vaccination with ChAdOx1 nCoV-19/AZD1222 the immune response persists for at least 12 months, and given the decay follows a non-linear trajectory, immunity is likely detectable for a significant period of time thereafter; modelling performed herein predicts a relatively high level of antibodies can be detected two years after vaccination. Given the ongoing transmission of SARS CoV-2, it is important to have a well-maintained immune response to protect against severe disease and minimize the burden on healthcare system.

## Supplementary Material

uxad013_suppl_Supplementary_Material

## Data Availability

Anonymized participant data will be made available when the trials are complete, upon requests directed to the corresponding author. Proposals will be reviewed and approved by the sponsor, investigator, and collaborators on the basis of scientific merit. After approval of a proposal, data can be shared through a secure online platform after signing a data access agreement.

## Abbreviations

AIC: Akaike’s Information Criteria
ELISA: enzyme-linked immunosorbent assay
ELISpot: enzyme-linked immunospot
EU: ELISA units
IgG: immunoglobulin G
LDLD: two low doses
LDSD: low dose followed by standard dose
mRNA: messenger ribonucleic acid
ND50: dilution to achieve 50% virus neutralization
SARS CoV-2: severe acute respiratory syndrome coronavirus 2
SDSD: 2 standard doses
SEAP: secreted embryonic alkaline phosphatase
UKHSA: United Kingdom Health Security Agency
VE: vaccine efficacy
VoC: variant(s) of concern

## Clinical trial registration

The clinical trials described in this manuscript are COV001, registered at clinicaltrials.gov as NCT04324606 and COV002 registered as NCT04400838.

## References

[1] Falsey AR , Sobieszczyk ME, Hirsch I, Sproule S, Robb ML, Corey L, et al. Phase 3 safety and efficacy of AZD1222 (ChAdOx1 nCoV-19) Covid-19 vaccine. N Engl J Med 2021, 385, 2348–60. doi:10.1056/NEJMoa2105290. 34587382 PMC8522798

[2] Voysey M , Clemens SAC, Madhi SA, Weckx LY, Folegatti PM, Aley PK, et al. Safety and efficacy of the ChAdOx1 nCoV-19 vaccine (AZD1222) against SARS-CoV-2: an interim analysis of four randomised controlled trials in Brazil, South Africa, and the UK. The Lancet 2021, 397, 99–111. doi:10.1016/S0140-6736(20)32661-1. PMC7723445 33306989

[3] Harris RJ , Hall JA, Zaidi A, Andrews NJ, Dunbar JK, Dabrera G. Effect of vaccination on household transmission of SARS-CoV-2 in England. N Engl J Med 2021, 385, 759–60. doi:10.1056/NEJMc2107717. 34161702 PMC8262621

[4] Hyams C , Marlow R, Maseko Z, King J, Ward L, Fox K, et al. Effectiveness of BNT162b2 and ChAdOx1 nCoV-19 COVID-19 vaccination at preventing hospitalisations in people aged at least 80 years: a test-negative, case-control study. Lancet Infect Dis 2021, 21, 1539–48. doi:10.1016/S1473-3099(21)00330-3. 34174190 PMC8221734

[5] Collie S , Champion J, Moultrie H, Bekker L-G, Gray GJNEJM. Effectiveness of BNT162b2 vaccine against omicron variant in South Africa. *NEJM* 2022, 386, 494–496. 10.1056/NEJMc2119270 PMC8757569 34965358

[6] UK Health Security Agency. SARS-CoV-2 variants of concern and variants under investigation in England. Technical briefing: update on hospitalisation and vaccine effectiveness for Omicron VOC-21NOV-01 (B.1.1.529). Investigation of SARS-CoV-2 variants: technical briefings UKHSA publications gateway number GOV-10920 (2021).

[7] Andrews N , Tessier E, Stowe J, Gower C, Kirsebom F, Simmons R, et al. Vaccine effectiveness and duration of protection of Comirnaty, Vaxzevria and Spikevax against mild and severe COVID-19 in the UK. *MedRxiv* (2021).

[8] Goldberg Y , Mandel M, Bar-On YM, Bodenheimer O, Freedman L, Haas EJ, et al. Waning immunity after the BNT162b2 vaccine in Israel. N Engl J Med 2021, 385, e85. doi:10.1056/NEJMoa2114228. 34706170 PMC8609604

[9] Doria-Rose N , Suthar MS, Makowski M, O'Connell S, McDermott AB, Flach B, et al. Antibody persistence through 6 months after the second dose of mRNA-1273 vaccine for Covid-19. N Engl J Med 2021, 384, 2259–61. doi:10.1056/NEJMc2103916. 33822494 PMC8524784

[10] Levin EG , Lustig Y, Cohen C, Fluss R, Indenbaum V, Amit S, et al. Waning immune humoral response to BNT162b2 Covid-19 vaccine over 6 months. N Engl J Med 2021, 385, e84. doi:10.1056/NEJMoa2114583. 34614326 PMC8522797

[11] Pan H , Wu Q, Zeng G, Yang J, Jiang D, Deng XE, et al. Immunogenicity and safety of a third dose, and immune persistence of CoronaVac vaccine in healthy adults aged 18-59 years: interim results from a double-blind, randomized, placebo-controlled phase 2 clinical trial. MedRxiv, 2021.2007.2023.21261026, doi:10.1101/2021.07.23.21261026 %J medRxiv (2021).

[12] Shaw RH , Liu X, Stuart ASV, Greenland M, Aley PK, Andrews NJ, et al. Effect of priming interval on reactogenicity, peak immunological response, and waning after homologous and heterologous COVID-19 vaccine schedules: exploratory analyses of Com-COV, a randomised control trial. Lancet Resp Med 2022, 10, 1049–1060. doi:10.1016/s2213-2600(22)00163-1. PMC9179150 35690076

[13] Ramasamy MN , Minassian AM, Ewer KJ, Flaxman AL, Folegatti PM, Owens DR, et al Safety and immunogenicity of ChAdOx1 nCoV-19 vaccine administered in a prime-boost regimen in young and old adults (COV002): a single-blind, randomised, controlled, phase 2/3 trial. The Lancet 2021, 396, 1979–93. doi:10.1016/S0140-6736(20)32466-1. PMC7674972 33220855

[14] Barrett JR , Belij-Rammerstorfer S, Dold C, Ewer KJ, Folegatti PM, Gilbride C, et al. Phase 1/2 trial of SARS-CoV-2 vaccine ChAdOx1 nCoV-19 with a booster dose induces multifunctional antibody responses. Nat Med 2021, 27, 279–88. doi:10.1038/s41591-020-01179-4. 33335322

[15] Folegatti PM , Ewer KJ, Aley PK, Angus B, Becker S, Belij-Rammerstorfer S, et al. Safety and immunogenicity of the ChAdOx1 nCoV-19 vaccine against SARS-CoV-2: a preliminary report of a phase 1/2, single-blind, randomised controlled trial. Lancet 2020, 396, 467–78. doi:10.1016/S0140-6736(20)31604-4. 32702298 PMC7445431

[16] Dicks MDJ , Spencer AJ, Edwards NJ, Wadell G, Bojang K, Gilbert SC, et al. A novel chimpanzee adenovirus vector with low human seroprevalence: improved systems for vector derivation and comparative immunogenicity. Plos One 2012, 7, e40385 . 22808149 10.1371/journal.pone.0040385 PMC3396660

[17] Marchevsky NG. An exploratory analysis of the response to ChAdOx1 nCoV-19 (AZD1222) vaccine in males and females. eBiomedicine 2022, 81, 104128 35779491 10.1016/j.ebiom.2022.104128 PMC9242842

[18] Collier AY , Yu J, McMahan K, Liu J, Chandrashekar A, Maron JS, et al. Differential kinetics of immune responses elicited by covid-19 vaccines. N Engl J Med 2021, 385, 2010–2. doi:10.1056/NEJMc2115596. 34648703 PMC8531985

[19] Cho A , Muecksch F, Wang Z, Tanfous BT, DaSilva J, Raspe R, et al. Antibody evolution to SARS-CoV-2 after single-dose Ad26.COV2.S vaccine in humans. J Exp Med 2022, 219: e20220732. doi:10.1084/jem.20220732. 35776090 PMC9253517

[20] Liu X , Munro APS, Feng S, Janani L, Aley PK, Babbage G, et al. Persistence of immunogenicity after seven COVID-19 vaccines given as third dose boosters following two doses of ChAdOx1 nCov-19 or BNT162b2 in the UK: three month analyses of the COV-BOOST trial. J Infect 2022, 84, 795–813. doi:10.1016/j.jinf.2022.04.018. 35405168 PMC8993491

[21] Wang Z , Muecksch F, Muenn F, Cho A, Zong S, Raspe R, et al. Humoral immunity to SARS-CoV-2 elicited by combination COVID-19 vaccination regimens. bioRxiv, 2022.2005.2013.491823, doi:10.1101/2022.05.13.491823 (2022). PMC9418484 36006380

[22] Payne RP , Longet S, Austin JA, Skelly DT, Dejnirattisai W, Adele S, et al. Immunogenicity of standard and extended dosing intervals of BNT162b2 mRNA vaccine. Cell 2021, 184, 5699–5714.e11. doi:10.1016/j.cell.2021.10.011. 34735795 PMC8519781

[23] Kaplonek, P, Fischinger S, Cizmeci D, Bartsch YC, Kang J, Burke JS, et al. mRNA-1273 vaccine-induced antibodies maintain Fc effector functions across SARS-CoV-2 variants of concern. Immunity 2022, 55, 355–365.e4. doi:10.1016/j.immuni.2022.01.001. 35090580 PMC8733218

[24] Tarke A , Coelho CH, Zhang Z, Dan JM, Yu ED, Methot N, et al. SARS-CoV-2 vaccination induces immunological T cell memory able to cross-recognize variants from alpha to omicron. Cell 2022, 185, 847–859.e11. doi:10.1016/j.cell.2022.01.015. 35139340 PMC8784649

