# Supplementary material for: Persistence of the immune response after two doses of ChAdOx1 nCov-19 (AZD1222): 1 year of follow-up of two randomized controlled trials
Source: Clin Exp Immunol. 2023 Feb 2;211(3):280–7. doi: 10.1093/cei/uxad013 (PMC10038323; doi:10.1093/cei/uxad013)
Supplement: uxad013_suppl_Supplementary_Material [file uxad013_suppl_supplementary_material.docx]

# Supplementary Material

**Figure S1 Anti-spike IgG by standardised ELISA with GMCs extrapolated to two years post vaccination**


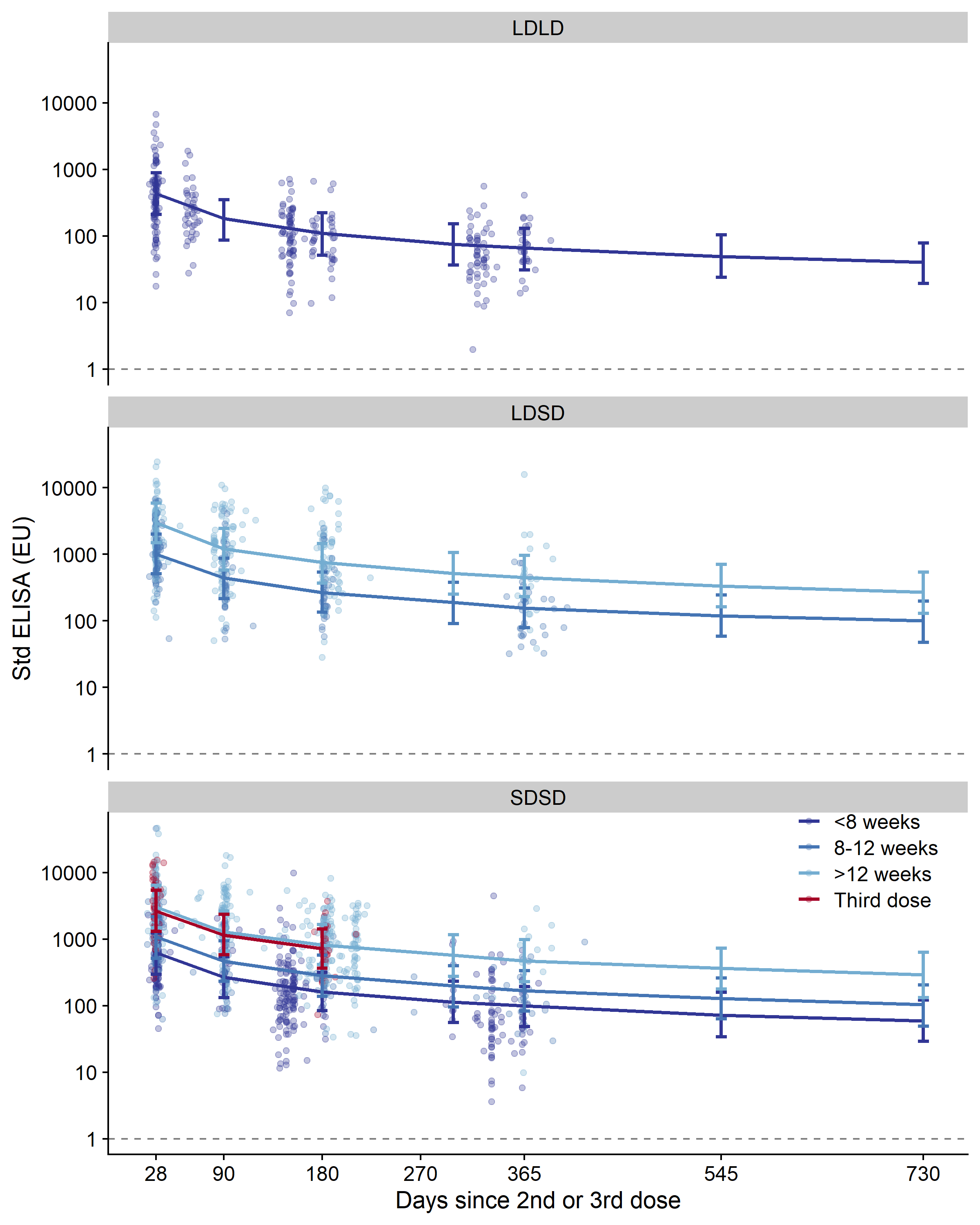


See Table S2 for values.

Table S1 Output from mixed effects regression model of log_10_-transformed anti-spike IgG titres shown in Figures 1 and 2

|  | Estimate | Std. Error | df | t value | Pr(>\|t\|) | Type 3 p value* |
| --- | --- | --- | --- | --- | --- | --- |
| (Intercept) | 3.8338 | 0.0458 | 1674.5 | 83.742 | < 0.0001 |  |
| Log_10_(days) | -0.718 | 0.015 | 1182.1 | -47.75 | < 0.0001 |  |
| interval: 8-12 weeks (ref <8 weeks) | 0.2398 | 0.0508 | 910.47 | 4.725 | < 0.0001 | <0.0001 |
| interval: >12 weeks (ref <8 weeks) | 0.689 | 0.042 | 1228.2 | 16.411 | < 0.0001 |  |
| vaccine_arm: LDLD (ref SDSD) | 0.6408 | 0.0451 | 1751.6 | 14.215 | 0.0006 | 0.0023 |
| vaccine_arm: LDSD (ref SDSD) | -0.176 | 0.0511 | 713.66 | -3.441 | 0.4652 |  |
| Age (per year) (centred on 40 years) | -0.031 | 0.0423 | 767.37 | -0.731 | 0.0008 |  |

* p value from Type III Wald chi square tests

Table S2 Model predicted geometric mean titres and confidence intervals from the regression model of anti-spike IgG shown in Figures 1 and 2 and Table S1

| **Days from boost** | **Vaccine arm** | **Vaccine Interval** | **GM** | **LCL** | **UCL** |
| --- | --- | --- | --- | --- | --- |
| 28 | LDLD | <8 weeks | 436 | 211 | 885 |
|  | SDSD | <8 weeks | 622 | 296 | 1228 |
|  | LDSD | 8-12 weeks | 1008 | 512 | 1993 |
|  | SDSD | 8-12 weeks | 1091 | 514 | 2165 |
|  | LDSD | >12 weeks | 2993 | 1483 | 5877 |
|  | SDSD | >12 weeks | 3020 | 1481 | 6398 |
| 90 | LDLD | <8 weeks | 183 | 87 | 348 |
|  | SDSD | <8 weeks | 268 | 131 | 541 |
|  | LDSD | 8-12 weeks | 443 | 215 | 871 |
|  | SDSD | 8-12 weeks | 466 | 231 | 936 |
|  | LDSD | >12 weeks | 1208 | 579 | 2443 |
|  | SDSD | >12 weeks | 1291 | 658 | 2629 |
| 180 | LDLD | <8 weeks | 110 | 52 | 222 |
|  | SDSD | <8 weeks | 162 | 84 | 318 |
|  | LDSD | 8-12 weeks | 265 | 136 | 543 |
|  | SDSD | 8-12 weeks | 285 | 139 | 576 |
|  | LDSD | >12 weeks | 761 | 367 | 1444 |
|  | SDSD | >12 weeks | 819 | 413 | 1657 |
| 300 | LDLD | <8 weeks | 75 | 37 | 153 |
|  | SDSD | <8 weeks | 113 | 56 | 233 |
|  | LDSD | 8-12 weeks | 190 | 91 | 377 |
|  | SDSD | 8-12 weeks | 198 | 95 | 396 |
|  | LDSD | >12 weeks | 517 | 253 | 1068 |
|  | SDSD | >12 weeks | 575 | 278 | 1172 |
| 365 | LDLD | <8 weeks | 66 | 31 | 130 |
|  | SDSD | <8 weeks | 99 | 48 | 194 |
|  | LDSD | 8-12 weeks | 155 | 79 | 313 |
|  | SDSD | 8-12 weeks | 167 | 83 | 335 |
|  | LDSD | >12 weeks | 447 | 232 | 967 |
|  | SDSD | >12 weeks | 472 | 228 | 978 |
| Extrapolated timepoints |  |  |  |  |  |
| 545 | *LDLD* | *<8 weeks* | *49* | *24* | *104* |
|  | *SDSD* | *<8 weeks* | *72* | *34* | *160* |
|  | *LDSD* | *8-12 weeks* | *119* | *59* | *246* |
|  | *SDSD* | *8-12 weeks* | *128* | *63* | *261* |
|  | *LDSD* | *>12 weeks* | *334* | *162* | *707* |
|  | *SDSD* | *>12 weeks* | *364* | *177* | *730* |
| 730 | *LDLD* | *<8 weeks* | *40* | *19* | *79* |
|  | *SDSD* | *<8 weeks* | *59* | *29* | *122* |
|  | *LDSD* | *8-12 weeks* | *100* | *47* | *197* |
|  | *SDSD* | *8-12 weeks* | *104* | *49* | *206* |
|  | *LDSD* | *>12 weeks* | *271* | *129* | *541* |
|  | *SDSD* | *>12 weeks* | *290* | *133* | *632* |

Table S3 Model predicted geometric mean titres and confidence intervals from the regression model of Interferon-γ ELISpot shown in Figure 3

| Days from boost | GM | 95% LCL | 95% UCL |
| --- | --- | --- | --- |
| 28 | 364 | 190 | 773 |
| 90 | 264 | 134 | 536 |
| 180 | 203 | 99 | 423 |
| 300 | 180 | 87 | 359 |
| 365 | 172 | 83 | 358 |

Table S4 Model predicted geometric mean titres and confidence intervals from the regression model of live virus microneutralisation titres shown in Figure 4

| Days from boost | GM | 95% LCL | 95% UCL |
| --- | --- | --- | --- |
| 28 | 455 | 196 | 1110 |
| 150 | 136 | 55 | 330 |
| 340 | 108 | 41 | 278 |

**Figure S2 Anti-ChAdOx1 vector neutralising titres**

**
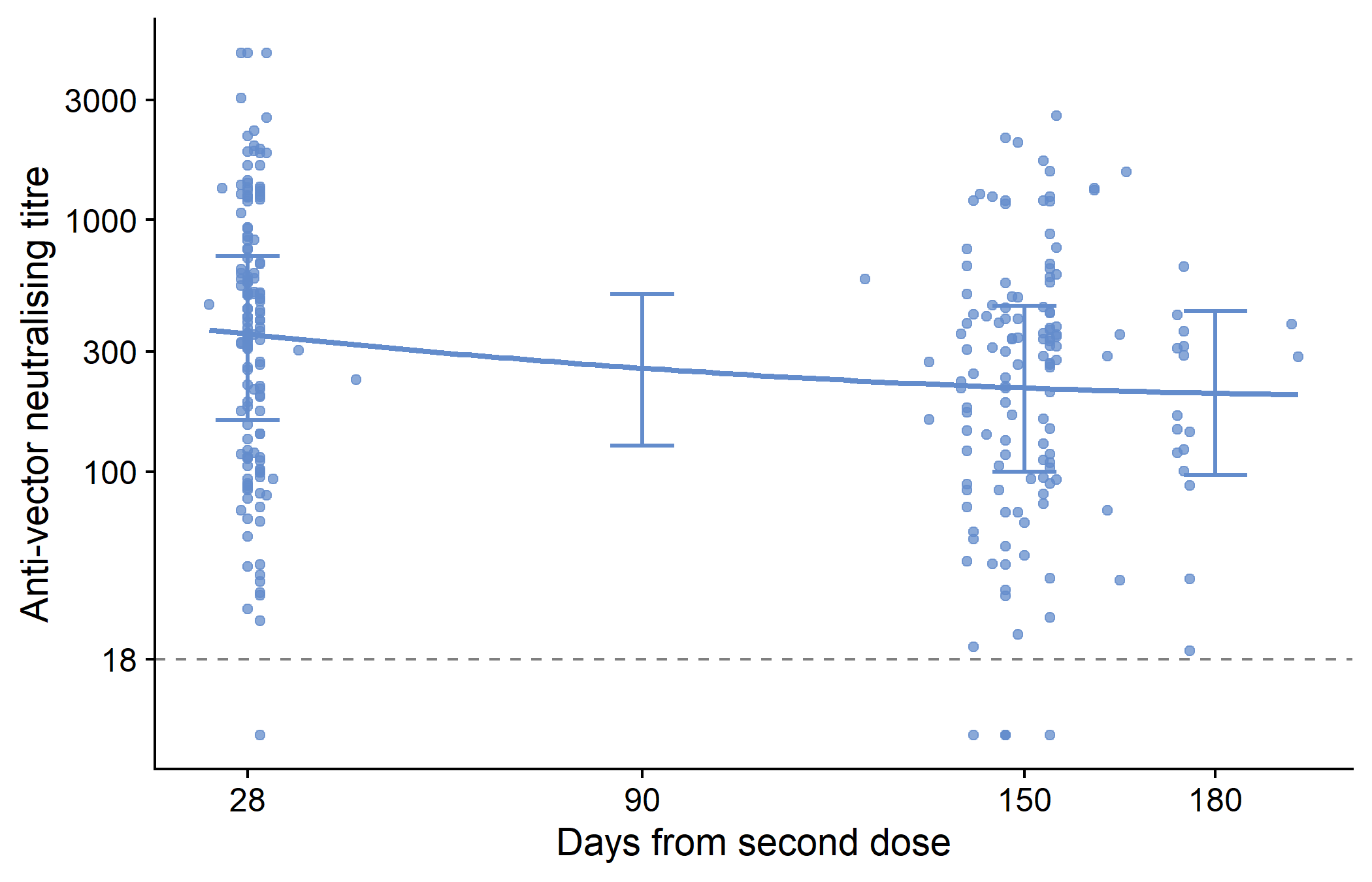
**

Table S5 Model predicted geometric mean titres and confidence intervals from the regression model of anti-vector neutralisation titres shown in Figure S2

| Days from boost | GM | 95% LCL | 95% UCL |
| --- | --- | --- | --- |
| 28 | 345 | 160 | 717 |
| 90 | 254 | 127 | 507 |
| 150 | 215 | 100 | 454 |
| 180 | 203 | 97 | 435 |
